# Blood *MAPT* expression and methylation status in Alzheimer's disease

**DOI:** 10.1002/pcn5.65

**Published:** 2022-12-14

**Authors:** Hiroaki Mori, Yuta Yoshino, Mariko Ueno, Yu Funahashi, Hiroshi Kumon, Yuki Ozaki, Kiyohiro Yamazaki, Shinichiro Ochi, Jun‐ichi Iga, Shu‐ichi Ueno

**Affiliations:** ^1^ Department of Neuropsychiatry, Molecules and Function Ehime University Graduate School of Medicine, Shitsukawa Toon Ehime Japan

**Keywords:** Alzheimer's disease, blood, gene expression, gene methylation, MAPT

## Abstract

**Aim:**

This study aimed to investigate the expression levels and methylation status of *microtubule‐associated protein tau* (*MAPT*) in the blood of Alzheimer's disease (AD) patients and age‐ and sex‐matched healthy controls.

**Methods:**

Fifty AD outpatients and 50 healthy contorls were enrolled. Blood samples were collected for processing of complementary DNA and genomic DNA. *MAPT* messenger ribonucleic acid (mRNA) expression was analyzed by real‐time quantitative polymerase chain reaction. The methylation rates of four cytosine‐phosphate‐guanine (CpG) sites in the upstream region of *MAPT* exon1 were evaluated by the pyrosequencing method.

**Results:**

No significant differences in *MAPT* mRNA expression levels were found between AD and control subjects (AD 0.97 ± 0.49 vs. control 1.0 ± 0.64, *p* = 0.62). *MAPT* mRNA expression levels were not correlated with any other clinical characteristics or results of psychological tests. *MAPT* mRNA expression levels were significantly higher in AD subjects treated with acetylcholinesterase inhibitors (AchEIs) (*n* = 25) than in subjects not treated with AChEIs (*n* = 25) (unmedicated 0.83 ± 0.33 vs. medicated 1.12 ± 0.59, *p* = 0.049). The AD subjects did not differ from the control subjects in methylation rates at selected CpG sites. *MAPT* methylation status were not correlated with clinical characteristics, the results of psychological tests, or *MAPT* mRNA expression.

**Conclusion:**

*MAPT* mRNA expression levels and methylation status in blood do not appear useful as biomarkers for AD or the examined CpG sites were not genetically significant for *MAPT* gene expression or AD pathology. However, AChEIs may alter *MAPT* mRNA expression. Further studies are needed to explore blood biomarkers that can discriminate AD patients from controls.

## Graphical abstract

MAPT mRNA expression levels and methylation status in blood do not appear useful as biomarkers for AD. However, acetylcholine esterase inhibitors may alter MAPT mRNA expression. *p < 0.05.

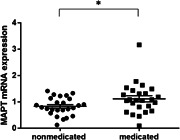

## INTRODUCTION

Alzheimer's disease (AD) is a chronic neurodegenerative disease that causes apraxia, aphasia, impairment of memory, and loss of motivation. Pathologically, it is characterized by senile plaques and neurofibrillary tangles.^1^ Senile plaques are extracellular accumulations of amyloid beta encoded by *amyloid precursor protein* on chromosome 21q21.^2^ Neurofibrillary tangles are the abnormal intracellular accumulation of the hyperphosphorylated protein tau encoded by *microtubule‐associated protein tau* (*MAPT*) on chromosome 17q21.^3^

*MAPT* has been associated with various neurodegenerative diseases. One of the most typical *MAPT*‐related disorders is frontotemporal dementia with parkinsonism linked to chromosome 17, which is caused by *MAPT* mutations.^4^ Progressive supranuclear palsy is also associated with the more common allele (H1) and genotype (H1H1) in *MAPT*.^5^

With regard to AD, increased *MAPT* messenger ribonucleic acid (mRNA) expression was found in the temporal lobe of sporadic AD patients,^6^ but no difference was found between AD and control subjects with regard to *MAPT* mRNA expression in peripheral blood mononuclear cells.^7^ The H2‐haplotype in *MAPT* has also been reported to reduce the risk of late‐onset AD,^8^ and rs393152, a single‐nucleotide polymorphism (SNP) at the *MAPT* locus, was reported to significantly increase the risk of AD.^9^

Changes in gene expression without changing DNA sequences are termed epigenetic. DNA methylation is one of several epigenetic processes that regulate gene expression^10^ and has been associated with the pathogenesis of various brain diseases.^11^, ^12^ In the promoter region of *MAPT*, the DNA methylation status of a cytosine‐phosphate‐guanine (CpG) island clearly affects mRNA expression.^6^, ^13^ In brains with sporadic AD, both neuronal and non‐neuronal cells had reduced methylation of this CpG island, suggesting that hypomethylation in AD patients increases tau expression, leading to tau aggregation and pathological spread throughout the brain, as in prion disease.^6^ In Parkinson's disease (PD) patients, *MAPT* hypermethylation has been reported to occur in the cerebellum, with hypomethylation in the putamen.^14^ On the other hand, it has been reported that methylation is not altered in AD, PD, and frontotemporal dementia.^15^

Currently, β‐amyloid (1–42), total tau, and phospho‐tau‐181 in cerebrospinal fluid are useful biomarkers for AD diagnosis.^16^ However, lumbar puncture is an invasive and expensive test, and minimally invasive biomarkers are needed that can differentiate AD patients from healthy controls. Our group has previously reported that the methylation status of *synuclein alpha* (*SNCA*) in blood could be a useful biomarker for AD.^17^ The involvement of immune function in the pathogenesis of AD has also been actively reported in recent years,^18^, ^19^, ^20^ attracting attention to blood biomarkers.^21^ We therefore hypothesized that *MAPT* mRNA expression levels and the methylation status in blood are biomarkers of AD. In particular, the methylation levels of *MAPT* in the blood of AD patients have not been investigated, therefore the expression levels and the methylation status of *MAPT* in blood of AD patients and age‐ and sex‐matched healthy controls were investigated.

## MATERIALS AND METHODS

### AD subjects and healthy control subjects

Demographic data for each group of participants are shown in Table 1. Fifty AD patients (17 males and 33 females, mean age ± standard deviation (SD) = 78.3 ± 6.2 years), who were outpatients at Ehime University Hospital and Zaidan Niihama Hospital, Ehime, Japan, were enrolled. AD subjects were diagnosed according to the Aging/Alzheimer's Association criteria.^22^ They were diagnosed as having probable AD and lived with at least one caregiver. Patients with AD without cerebrovascular lesions on head computed tomography or magnetic resonance imaging were included. AD subjects were assessed with the Mini‐Mental State Examination (MMSE) and Alzheimer's Disease Assessment Scale (ADAS), which assesses cognitive functions,^23^, ^24^ the Montgomery‐Åsberg Depression Rating Scale (MADRS) to assess depression symptoms,^25^ Clinical Dementia Rating (CDR) by family caregivers,^26^ and the Neuropsychiatric Inventory (NPI)^27^ to assess psychological symptoms.

**Table 1:** Alzheimer's disease and control subjects

	AD subjects	Control subjects	*P* value
*N*	50	50	
Sex (male:female)	17:33	11:39	0.18
Age (y)	78.3 ± 6.2	76.2 ± 6.2	0.10
ApoE ε4+/ε4–	27/23	7/43	<0.001

*Note*: Values are the mean ± standard deviation.

Abbreviations: AD, Alzheimer's disease; ApoE, apolipoprotein E.

Controls were 50 elderly people (11 males and 39 females, mean age ± SD = 76.2 ± 6.2 years) with no cognitive impairment, psychiatric symptoms, or history of previous mental disorders and diagnosed as mentally and cognitively normal by at least two certified psychiatrists based on clinical interviews. All participants were unrelated Japanese and provided written, informed consent approved by the institutional ethics committees of Ehime University Hospital and Zaidan Niihama Hospital (approval numbers: 31‐K8, 1901009, and 2109001).

### Blood sample collection and processing of cDNA and gDNA

Total RNA was collected from whole peripheral blood samples into PaxGene Blood RNA Systems tubes (BD) and extracted according to the manufacturer's protocol. The RNA concentration and purity were measured on a NanoDrop‐1000 (Thermo Fisher Scientific) system, with acceptable 260/280 ratios in the range of 1.8–2.0. For each 40‐µl reaction, complementary DNA (cDNA) was synthesized with the High‐Capacity cDNA Reverse Transcription Kit (Applied Biosystems) with 1.0 µg of RNA as template. The genomic DNA (gDNA) samples were obtained from whole peripheral blood samples collected in potassium ethylenediaminetetraacetic acid tubes. The gDNA was extracted according to the manufacturer's protocol using the QIAamp DNA Blood Mini Kit (Qiagen).

### PCR procedure

Expression of mRNA was analyzed by real‐time quantitative polymerase chain reaction (PCR) using a StepOnePlus Real‐Time PCR System (Applied Biosystems). The specific Taq‐Man probes were Hs00902193 m1 for *MAPT* and Hs99999905 m1 for *GAPDH* (Applied Biosystems). *GAPDH* is a suitable housekeeping gene for quantitative RT‐PCR and was used as the reference gene in this study.^28^, ^29^, ^30^ The final volume of each reaction containing TaqMan Universal Master Mix (Applied Biosystems) was 10 µl. Expression levels were examined by duplicate measurements. The ΔΔ*C*_t_ method and StepOne software (Applied Biosystems) were used to measure relative expression levels.

### Bisulfite conversion and pyrosequencing

**Figure 1:**
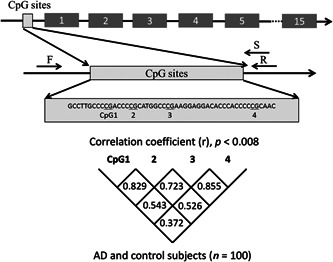
Schematic diagram showing the location of *MAPT*. Correlations between pairs of the four CpGs were analyzed with Spearman's rank correlation coefficient. Statistical significance was defined at *P* = 0.008 (= 0.05/6) following Bonferroni correction. F, forward primer; R, reverse primer; S, sequence primer; AD, Alzheimer's disease subjects; CpG, cytosine‐phosphate‐guanine.

In pyrosequencing, we analyzed the upstream region of *MAPT* exon1, which was predicted as a CpG island using the Sequence Manipulation Suite (https://www.bioinformatics.org/sms2/index.html) (Figure 1). The primers of pyrosequencing were designed using PyroMark Assay Design Software (Qiagen). The gDNA (1000 ng/sample) extracted from whole blood was converted with bisulfate using the EpiTect Plus DNA Bisulfite Kit (Qiagen). PCR amplification was then performed using forward primer (5‐TGGAAGGTAGTTTAGGATTTTTGTAGG‐3) and reverse primer (5‐[Biotin]‐CCACCTCCTATAATTAAAATCTTTAT‐3) with the converted gDNA as template. The final volume of each PCR reaction was 15.0 μl, and each reaction contained 1.2 μl of gDNA, 0.2 µM forward and reverse primers, 0.5 U AmpliTaq gold (Applied Biosystems), 10× PCR buffer with 15 mM MgCl_2_, and 2 mM dNTP. Cycling conditions were denaturation for 10 min at 94°C, 45 cycles of 95°C (30 s), 54°C (30 s), and 72°C (1 min), with final extension at 72°C (5 min). PCR products were sequenced using PyroMark Q24 Advanced (Qiagen) and sequencing primer, 5‐CTCCTATAATTAAAATCTTTATATC‐3. The methylation rate of each CpG site was quantified in duplicate using PyroMark Q24 Advanced Software (Qiagen).

### Genotyping

Using a StepOnePlus real‐time PCR system (Applied Biosystems), we conducted genotyping of SNPs (rs429358 and rs7412) in *APOE* with a TaqMan 5′‐exonuclease allelic discrimination assay (Assay ID: rs429538; C__3084793_20, rs7412; C__904973_10; Applied Biosystems). The *APOE* ε2, ε3, and ε4 alleles are derived from the two genotypes of rs429358 (T334C) and rs7412 (C472T): ε2; 334T/472T, ε3; 334T/472C, and ε4; 334C/472C. The ε4 genotype is associated with an increased risk for AD.^31^

### Statistical analysis

Statistical analyses were performed using SPSS 22.0 software (IBM Japan). Sex and *APOE* genotype were analyzed by Fisher's exact test. The Shapiro–Wilk test was used to test for normality. Comparisons of *MAPT* mRNA expression and each CpG methylation rate in AD and control subjects were performed by the Mann–Whitney *U* test. Comparisons of *MAPT* mRNA expression and each CpG methylation rate by sex, *APOE* genotype, and use of acetylcholinesterase inhibitors (AChEIs) were also performed using the Mann–Whitney *U* test. Correlations of *MAPT* mRNA expression with age at blood sampling, age of onset, duration of disease, education, MMSE, ADAS, NPI, MADRS, CDR, the average of four CpG methylation rates, and methylation rate at each CpG site were performed using Spearman's rank correlation coefficient. Correlations between pairs of the four CpGs methylation rates were also analyzed with Spearman's rank correlation coefficient. Correlations of the average of four CpG methylation rates with age at blood sampling, age of onset, duration of disease, education, MMSE, NPI, ADAS, MADRS, and CDR were also performed using Spearman's rank correlation coefficient. Significance was defined at the 95% level (*P* = 0.05). Statistical significance of correlations between pairs of the 4 CpGs with Spearman's rank correlation coefficient was defined at *P* = 0.008 (= 0.05/6) following Bonferroni correction. Statistical significance of correlations *MAPT* mRNA expression with the clinical characteristics was defined at *P* = 0.004 (= 0.05/14) following Bonferroni correction. Statistical significance of correlations the average of four CpG methylation rates with the clinical characteristics was defined at *P* = 0.006 (= 0.05/9) following Bonferroni correction.

## RESULTS

### Participant characteristics

Demographic data and *APOE* genotypes of the participants are presented in Table 1. There were no differences in sex (*P* value = 0.18) and age (*P* value = 0.10) between AD and control subjects. However, there was a significant difference in *APOE* genotype between the AD and control subjects (*P* value < 0.001). Clinical characteristics of AD subjects and results of psychological tests are presented in Table 2.

**Table 2:** Demographic and clinical data of Alzheimer's disease subjects

Characteristic	Alzheimer's disease subjects	*N*
Sex (male/female)	17/33	50
Age (y)	78.3 ± 6.2	50
Age at onset (y)	73.5 ± 6.5	50
Duration of illness (y)	5.4 ± 4.5	41
Education (y)	11.0 ± 2.8	47
Cholinesterase inhibitor medication (%)	50	50
MMSE total score	19.2 ± 6.7	50
ADAS total score	17.1 ± 9.0	34
NPI‐10 total score	13.3 ± 16.1	47
MADRS total score	6.3 ± 5.3	34
CDR score (0/0.5/1/2/3)	0/11/19/13/3	46

*Note*: Values are means ± standard deviation.

Abbreviations: ADAS, Alzheimer's Disease Assessment Scale; CDR, Clinical Dementia Rating; MADRS, Montgomery‐Asberg Depression Rating Scale; MMSE, Mini‐Mental State Examination; NPI, neuropsychiatric inventory.

### *MAPT* mRNA expression levels

**Figure 2:**
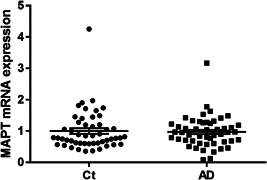
*MAPT* mRNA expression levels in AD and control subjects. The mean expression level in AD subjects (average ± SD = 0.97 ± 0.49) is not different from that in control subjects (average ± SD = 1.0 ± 0.64) (Mann–Whitney *U* test, *P* = 0.62). The horizontal bars represent the means ± standard error. AD, Alzheimer's disease subjects; Ct, control subjects

According to the Shapiro–Wilk test, *MAPT* expression levels were not normally distributed in both AD group and control subjects. No differences in *MAPT* mRNA expression levels were found between AD and control subjects (AD 0.97 ± 0.49 vs. control 1.0 ± 0.64, *P* value = 0.62; Figure 2).

**Figure 3:**
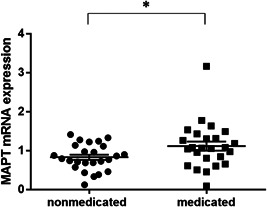
*MAPT* mRNA expression levels in AD subjects treated with acetylcholinesterase inhibitors (AChEIs) (*n* = 25) and those not treated with AChEIs (*n* = 25). The mean expression level in nonmedicated subjects is significantly different from that in medicated subjects (nonmedicated: 0.83 ± 0.33 vs. medicated: 1.12 ± 0.59, *P* = 0.049). The horizontal bars represent the means ± standard error. *p < 0.05.

*MAPT* mRNA expression levels were not significantly different between males and females (AD *P* value = 0.23, Cnt *P* value = 0.10) or between ApoE ε4+ and ε4− (AD *P* value = 0.34, Cnt *P* value = 0.48). *MAPT* mRNA expression levels were significantly higher in AD subjects treated with AChEIs (*n* = 25) than in subjects not treated with AChEIs (*n* = 25) (unmedicated 0.83 ± 0.33 vs. medicated 1.12 ± 0.59, *P* value = 0.049; Figure 3). Between unmedicated AD and control subjects, no significant difference in *MAPT* mRNA expression was found (*P* value = 0.58). *MAPT* mRNA expression levels were not correlated with age at blood sampling (*ρ* = −0.02, *P* value = 0.89), age of onset (*ρ* = −0.23, *P* value = 0.16), duration of disease (*ρ* = 0.25, *P* value = 0.13), or education (*ρ* = 0.06, *P* value = 0.71), ADAS (*ρ* = 0.20, *P* value = 0.25), NPI (*ρ* = −0.02, *P* value = 0.99), MADRS (*ρ* = 0.15, *p*‐value = 0.40), CDR (*ρ* = 0.04, *P* value = 0.79), and MMSE total score (*ρ* = 0.92, *P* value = 0.53) in all AD patients. MAPT mRNA expression levels were not correlated with blood sampling (*ρ* = −0.11, *P* value = 0.61), age of onset (*ρ* = −0.71, *P* value = 0.77), duration of disease (*ρ* = −0.17, *P* value = 0.50), or education (*ρ* = 0.24, *P* value = 0.27), ADAS (*ρ* = −0.07, *P* value = 0.78), NPI (*ρ* = −0.13, *P* value = 0.55), MADRS (*ρ* = −0.23, *P* value = 0.36), CDR (*ρ* = −0.21, *P* value = 0.33), and MMSE total score (*ρ* = 0.35, *P* value = 0.08) in unmedicated AD patients.

### Methylation status of *MAPT*

**Figure 4:**
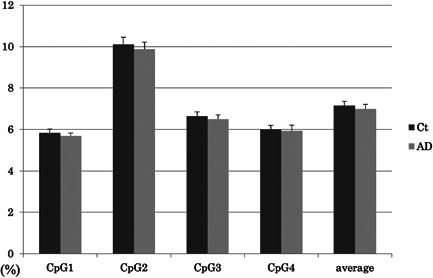
*MAPT* methylation rates in AD and control subjects at each CpG site. The values are the mean methylation rates + standard error. Ct, control subjects; AD, Alzheimer's disease subjects

The four target CpG sites in the upstream region of *MAPT* exon 1 are shown in Figure 1. The methylation rates of *MAPT* at each CpG site in AD and control subjects are shown in Figure 4. According to the Shapiro–Wilk test, the methylation rates of *MAPT* at each CpG site and the average rate of all CpG sites were not normally distributed in both AD group and control subjects except for CpG4 methylation rates in the healthy control group.

The AD group did not differ from the control group in methylation rates at CpG1 (AD vs. control subjects mean ± SD = 5.68 ± 1.11 vs. 5.83 ± 1.27, *P* value = 0.47), CpG2 (9.87 ± 2.44 vs. 10.10 ± 2.48, *P* value = 0.51), CpG3 (6.51 ± 1.40 vs. 6.65 ± 1.37, *P* value = 0.47), CpG4 (5.93 ± 2.00 vs. 6.00 ± 1.33, *P* value = 0.33), and average (6.99 ± 1.54 vs. 7.15 ± 1.47, *P* value = 0.41). Between the medicated AD and nonmedicated AD groups, no significant differences in methylation rate of each CpG site and the average rate of all sites were found. In addition, no significant differences in methylation rate of each CpG site and the average rate of all sites were found between unmedicated AD and control participants.

In all participants, the methylation rate of each CpG site and the average rate of all sites were not correlated with the expression of *MAPT* mRNA. In the nonmedicated AD and healthy control groups, no correlation was observed between *MAPT* mRNA expression and methylation status of each CpG site and the average rate of all sites. The average rate of all CpG sites was not significantly different between males and females (AD *P* value = 0.34, Cnt *P* value = 0.34) or between ApoE ε4+ and ε4– (AD *P* value = 0.51, Cnt *P* value = 0.76). The average rate of all CpG sites was correlated with age of onset (*ρ* = 0.32, *P* value = 0.05), ADAS score (*ρ* = −0.35, *P* value = 0.02), and MMSE total score (*ρ* = 0.30, *P* value = 0.03), although the significance disappeared after Bonferroni correction. The average rate of all CpG sites was not correlated with age at blood sampling (*ρ* = 0.28, *P* value = 0.05), duration of disease (*ρ* = −0.27, *P* value = 0.10), education (*ρ* = −0.15, *P* value = 0.34), NPI (*ρ* = −0.27, *P* value = 0.07), MADRS (*ρ* = −0.10, *P* value = 0.57), CDR (*ρ* = −0.35, *P* value = 0.79), and MMSE total score (*ρ* = 0.30, *P* value = 0.03) in all AD patients.

The average rate of all CpG sites was correlated with age at blood sampling (*ρ* = 0.43, *P* value = 0.03), although the significance disappeared after Bonferroni correction. The average rate of all CpG sites was not correlated with age of onset (*ρ* = 3.67, *P* value = 0.12), duration of disease (*ρ* = −0.32, *P* value = 0.19), education (*ρ* = −0.37, *P* value = 0.08), ADAS (*ρ* = −0.28, *P* value = 0.28), NPI (*ρ* = −0.34, *P* value = 0.12), MADRS (*ρ* = −0.04, *P* value = 0.89), CDR (*ρ* = −0.12, *P* value = 0.60), and MMSE total score (*ρ* = 0.20, *P* value = 0.35) in unmedicated AD subjects.

## DISCUSSION

The present study had three major findings.

First, blood *MAPT* mRNA expression levels and the methylation status did not differ between AD and control subjects. In addition, *MAPT* expression was not correlated with the ε4 genotype of APOE, which is a genetic risk for developing AD.^31^ Increased *MAPT* mRNA expression and hypomethylation of the CpG islands have been reported in the temporal lobe of AD patients.^6^ This may be due to the different expression levels and methylation status of *MAPT* in the brain and the blood. Tau proteins are mainly expressed in neurons, and the amount of *MAPT* expression in blood is much lower than in the brain.^32^ The methylation status of the CpG islands targeted in this study did not correlate with the expression levels of *MAPT* mRNA. Further studies are needed to identify the CpG regions that affect the expression levels and to determine the differences in *MAPT* expression levels and methylation status between brain and blood.

Second, *MAPT* mRNA expression levels were higher in AD subjects treated with AChEIs than in those who were not. This result suggests that the use of AChEIs may affect *MAPT* expression in AD patients. It has been reported that AChEIs have anti‐inflammatory effects.^33^, ^34^ A previous study reported that AChEIs suppressed microglial activation, neuronal loss, and tau pathology in tauopathy mouse model.^35^ On the other hand, anticholinergic activity accelerated the pathology of neurodegeneration with increased inflammation.^36^ In this study, it was suggested that change in *MAPT* expression in the blood of AD subjects with AChEIs reflects the effect of the medication on ameliorating tau pathology. However, the metabolic mechanisms of tau are not fully understood,^37^ and further studies are needed to determine whether or not AChEIs affect *MAPT* expression levels in the blood and the brain.

Third, the average rate of all CpG sites was correlated with age of onset, ADAS score, and MMSE total score, although the significance disappeared after Bonferroni correction. This result can be interpreted as showing that the increased average levels of CpG sites might be associated with higher MMSE and lower ADAS scores and may delay the age of disease onset. Thus, further studies are needed to analysis methylation status in the other CpG sites of the *MAPT* promoter region.

There are several potential weaknesses in this study. First, the relatively small sample size may lead to type II error. Second, brain–blood correlations in *MAPT* mRNA expression and methylation status were not examined. This information could shed light on the mechanisms by which epigenetic control regulates the expression levels of *MAPT* mRNA in the blood. *MAPT* mRNA expression levels and methylation status in the blood did not differ between AD and controls, suggesting that they cannot be used as biomarkers for AD. Studies are needed to explore blood biomarkers that are less invasive and can discriminate AD patients from controls.

## AUTHOR CONTRIBUTIONS

H.M., Y.Y., J.I., and S.U. designed the research. Y.Y., M.U., Y.F., H.K., Y.O., K.Y., and S.O. contributed to the accumulation of the data. H.M. wrote the manuscript. Y.Y., J.I., and S.U. contributed to the critical revision of the manuscript; All authors approved the final version of manuscript.

## CONFLICT OF INTEREST

The authors have no conflict of interest to declare.

## ETHICS APPROVAL STATEMENT

All participants were unrelated, of Japanese origin, and provided written, informed consent using forms that were approved by the institutional ethics committees of each hospital and of Ehime University Hospital (31‐K8, 1901009 and 2109001).

## PATIENT CONSENT STATEMENT

All participants provided written informed consent for publication of this paper.

## CLINICAL TRIAL REGISTRATION

N/A.

## Supporting information

Supplementary information.

## Data Availability

The raw data can be downloaded as supplementary data. Requests for other information should be sent to the corresponding author, Jun‐ichi Iga (Neuropsychiatry, Molecules and Function, Ehime University Graduate School of Medicine, Shitsukawa, Toon, Ehime, Japan; igajunichi@hotmail.com).
